# Vedolizumab for acute gastrointestinal graft-versus-host disease: A systematic review and meta-analysis

**DOI:** 10.3389/fimmu.2022.1025350

**Published:** 2022-11-11

**Authors:** Allen Cheng-Wei Li, Chen Dong, Soon-Tzeh Tay, Ashwin Ananthakrishnan, Kevin Sheng-Kai Ma

**Affiliations:** ^1^ School of Medicine, Chung Shan Medical University, Taichung, Taiwan; ^2^ Department of Medical Education, Chung Shan Medical University Hospital, Taichung, Taiwan; ^3^ Department of Medical Education, Chang Gung Memorial Hospital, Taoyuan, Taiwan; ^4^ Division of Gastroenterology, Massachusetts General Hospital and Harvard Medical School, Boston, MA, United States; ^5^ Department of Epidemiology, Harvard T.H. Chan School of Public Health, Boston, MA, United States; ^6^ Department of Dermatology, Massachusetts General Hospital, Boston, MA, United States; ^7^ Center for Global Health, Perelman School of Medicine, University of Pennsylvania, Philadelphia, PA, United States

**Keywords:** vedolizumab, graft-versus-host disease, biologics, alpha-4-beta-7 integrins, acute GVHD

## Abstract

**Objective:**

To determine the safety and efficacy of vedolizumab for the prophylaxis and treatment of gastrointestinal involvement of acute graft-versus-host disease (GVHD) (GI-aGVHD).

**Methods:**

Literature search within PubMed, EMBASE, Web of Science, and Cochrane Library for observational studies and clinical trials that evaluated the effect of vedolizumab on GI-aGVHD was done through 17 May 2022. A bivariate and random-effect meta-analysis derived the pooled observational percentages and pooled risk ratios (RRs) from baseline of primary endpoints including overall response, complete response, mortality, and adverse events.

**Results:**

There was a total of 122 participants in eight eligible studies, including one study on the prophylactic use of vedolizumab and seven studies on vedolizumab for the treatment of GI-aGVHD. Of seven studies that reported details on baseline grades of GI-aGVHD, a total of 47 patients (47.95%) were of stage 4, 31 patients (31.63%) were of stage 3, 10 patients (10.2%) were of stage 2, and 10 patients (10.2%) were of stage 1. The use of vedolizumab for the treatment of GI-aGVHD yielded a significantly improved objective response rate (ORR) at 14 days (pooled ORR = 60.53%, pooled RR = 14.14, 95% CI: 2.95–67.71), 28 days (pooled ORR = 50%, RR = 7.36, 95% CI = 2.14–25.37), and 12 months (pooled ORR = 76.92%, RR = 13.66, 95% CI = 3.5–53.35) from baseline. Likewise, the use of vedolizumab was followed by a significantly improved complete response (CR) at 12 months (pooled CR = 27.27%, RR = 5.50, 95% CI = 1.01–29.95), yet the CR at 14 days and 28 days did not reach statistical significance. Fifty-seven out of 87 (pooled overall survival, OS = 34.5%) and 46 out of 65 (pooled OS = 29.2%) patients expired at 6 and 12 months after the use of vedolizumab, respectively. Prophylactic use of vedolizumab was not associated with any specific type of reported adverse events, while patients with GI-aGVHD on vedolizumab presented with significantly increased risks of adverse events including infections (RR = 7.55) and impaired metabolism or nutritional complications (RR = 9.00). All analyses were of a low heterogeneity (all I-squares = 0%).

**Conclusion:**

Vedolizumab was safe and effective for the prophylaxis and management of early grade GI-aGVHD. More clinical evidence is warranted to validate these findings.

**Systematic review registration:**

https://www.crd.york.ac.uk/prospero/display_record.php?RecordID=345584, identifier CRD42022345584.

## Introduction

Hematopoietic cell transplantation (HSCT) is a medical procedure in which stem cells are infused into patients following short-term courses of chemotherapy or radiotherapy for hematopoietic malignancies or benign hematologic conditions (1, 2). Despite its success, severe graft-versus-host disease (GVHD) as a result of HSCT remains an unmet medical challenge, whose incidence ranges from 20% to 80% (3). The mechanisms by which acute GVHD occurs involve donor-activated T cells that recognize the recipient as foreign, which initiate immune reactions in various organs including skin, liver, lungs, and the gastrointestinal (GI) tract; as such, immunosuppressive medications are used for its prophylaxis (4). aGVHD is staged from grades I to IV according to the extent of organ involvement and the number of involved organs. Common risk factors for aGVHD include unrelated donors, mismatched donors, graft types, multiparous female donors, older ages of donors, and recipients (4). Although skin is the most common affected organ, cumulative evidence from previous studies has suggested the GI involvement of aGVHD (GI-aGVHD), manifested as persistent anorexia, diarrhea, abdominal pain, and hemorrhage, that is associated with great all-cause mortality (70%–90%) (5–7).

Existing options for the first-line treatment of grade II or above aGVHD other than corticosteroids are limited, with the compromised efficacy of corticosteroids resulting in fewer than 50% of patients having durable remission following treatments that only involved corticosteroids (8). For this, several combinations of steroids and additional systemic therapies have been used to treat steroid-refractory (SR) aGVHD (SR-aGVHD), although significant adverse events are as well noted. For instance, despite previous studies on cyclosporine, mycophenolate mofetil (MMF), ruxolitinib, extracorporeal photopheresis (ECP), tacrolimus, sirolimus, basiliximab, daclizumab, and infliximab have demonstrated the efficacy of these agents for GI symptoms of aGVHD, long-term use of these immunosuppressive agents is associated with high infection rate and poor life quality (9–11). As such, patients suffering from GI-aGVHD remained of poor prognosis and survival due to a lack of management that allows for both high treatment response and low infectious adverse events, following which damage to GI tract amplifies the severity of aGVHD through microbiota-triggered activation of inflammatory pathways (12, 13).

Vedolizumab, a monoclonal antibody that inhibits the interaction between α4β7 integrins on T lymphocytes and mucosal vascular addressin cell adhesion molecule 1 (MADCAM1) on gut endothelial cells, was reported to exert favorable efficacy and safety profile in bio-naïve patients with inflammatory bowel disease (IBD) including both ulcerative colitis and Crohn’s disease (14, 15) through blocking the homing of TH1, TH2, TH17, and Treg cells to inflamed colon and their subsequent accumulation in the tissue. The activation of α4β7 integrins by antigen-presenting cells (APC) in the GI tract is crucial for the trafficking of immunocompetent donor T lymphocytes to GI mucosa and gut-associated lymphoid tissues (GALT), which is also involved in the pathogenesis of GI-aGVHD (16, 17). However, clinical studies on vedolizumab for the treatment of GI-aGVHD were of limited sample sizes, with each studies typically enrolling less than 30 participants; thus, the efficacy cannot be robustly concluded (18). Moreover, there is a lack of studies on whether vedolizumab may be used as standard prophylaxis for GI-aGVHD in patients undergoing HSCT (19). Thus, the present systematic review and meta-analysis was designed to determine the safety and efficacy of vedolizumab for the prophylaxis and treatment of GI-aGVHD, respectively.

## Materials and methods

### Literature search

Studies on vedolizumab for the management of GI-aGVHD were retrieved and reviewed. We searched the following databases: MEDLINE, EMBASE, Web of science, and the Cochrane Library on May 17th, 2022 in accordance with the Preferred Reporting Items for Systematic Review and Meta-Analyzes (PRISMA) statement (20). The search strategy was developed using a combination of keywords as follows: (“biologics” OR “biological” OR “vedolizumab” OR “α4β7” OR “alpha4beta7”) AND (“graft versus host disease” OR “graft-versus-host disease” OR “gvhd”). We manually searched for further references to included research that were relevant.

### Study selection

**Figure 1:**
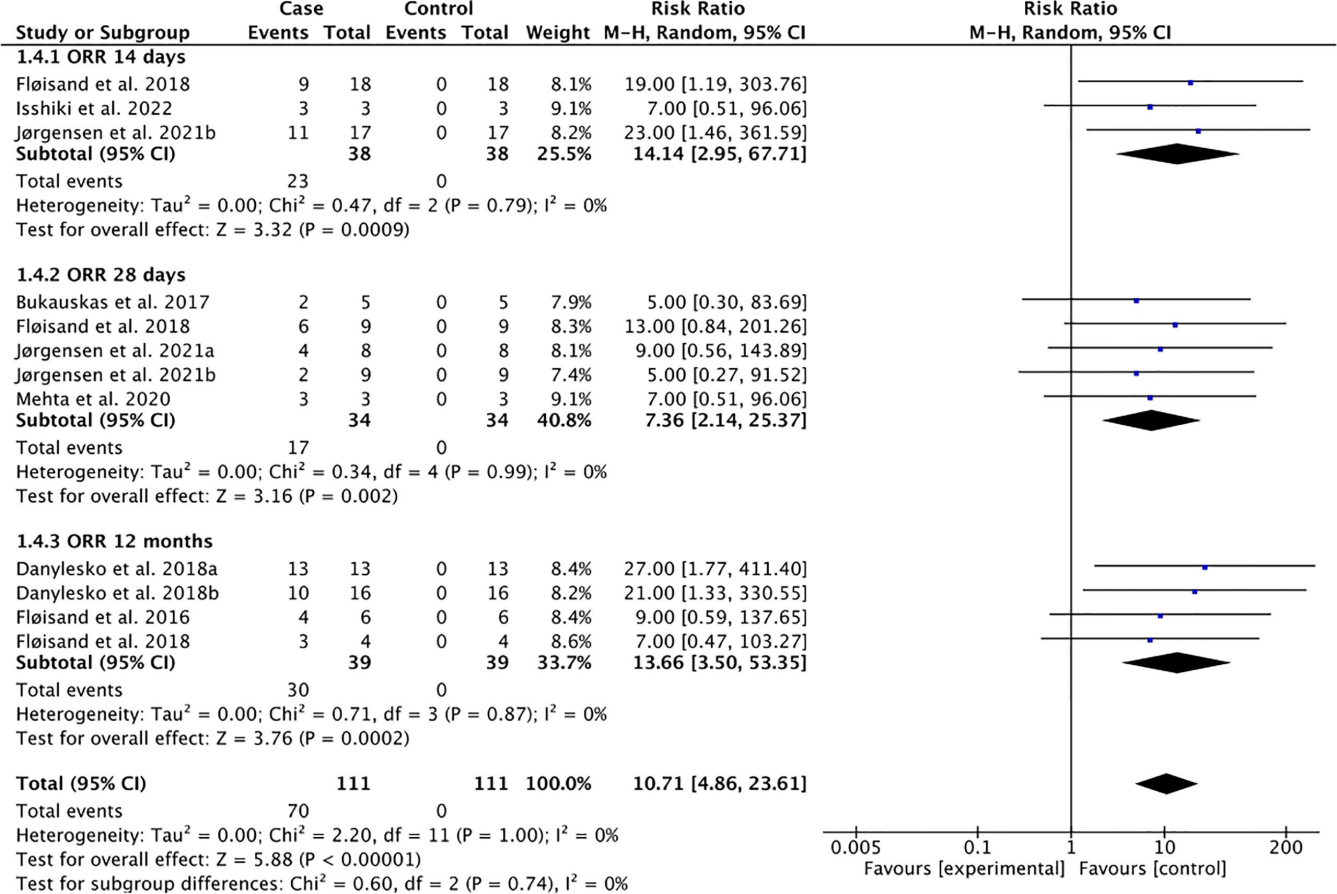
Overall response rate (ORR) following the use of vedolizumab for GI-aGVHD.

Two investigators extracted all data independently to ensure accuracy and consistency. Disagreements were settled by consensus or by seeking an independent third viewpoint (21–48). Eligible studies were clinical studies on vedolizumab for GVHD including either aGVHD or chronic GVHD (cGVHD). Letters to editor, expert comments, and studies without recorded treatment course were excluded (**Figure 1**).

### Screening and quality assessment

Two reviewers independently selected studies by screening titles and abstracts to identify those potentially relevant to our study question. Reported results of the included studies were extracted and analyzed. Disagreement was settled by discussion and review of the articles. The quality of included studies was assessed according to the Newcastle–Ottawa Scale (NOS) and NOS modified for single-arm cohorts (25). The ratings were made based on the quality of selection (up to four points), comparability (up to two points), and outcome of study participants (up to three points). The overall quality of study was defined as poor (scores 0–3), fair (scores, 4–6), or good (scores, 7–9). The quality assessment was carried out independently by two investigators. If there was a disagreement, it was resolved through discussion.

### Data extraction

Data on baseline demographics including country in which the study was conducted, study population, age, sex, and disease characteristics for the indication of HSCT, the grade and organs involved in aGVHD, and the usage, dose, intervention time, and duration of treatment of vedolizumab were extracted. Outcomes recorded during the follow-up period included main treatment response such as overall response, complete response, mortality, skin involvement, intestinal involvement, and hepatic involvement. Specifically, partial response was defined as an improvement of one aGVHD grade in at least one organ without progression in any other organs, while complete response was defined as the resolution of all signs and symptoms of aGVHD, according to the criteria described by Martin et al. (26). Overall response rate (ORR) included partial response rate plus complete response rate (CRR), as obtained on day 14, day 28, and 12 months after the initiation of vedolizumab treatment. Adverse events related to vedolizumab were also recorded.

### Statistical analysis and meta-analysis

A bivariate meta-analysis was used to derive the pooled estimates of outcomes including overall response, complete response, mortality, and adverse events following the use of vedolizumab in patients with GI-aGVHD, measured as pooled observational percentages of all participants and pooled Mantel–Haenszel (M–H) risk ratios with 95% confidence intervals (CIs) in the random-effect model using RevMan5 software (Cochrane Collaboration). For single-arm studies, the outcomes were compared with that at baseline, with which all outcomes at day 0 were set as zero. I-square was derived to determine the heterogeneity of all analyses, with I-square values less than 50% indicating between-study homogeneity. A p-value less than 0.05 in tests for overall effect indicated significant differences after the use of vedolizumab from baseline.

## Results

A total of 7,432 studies were identified through initial search. After screening the studies with titles and abstracts, 98 studies were further assessed, during which 90 studies were excluded due to duplicated study population or not human subjects research. Eight studies met the inclusion criteria (**Figure S1**), for which the study design and primary outcomes were summarized (**Table 1**).

**Table 1:** Overview of studies included in the systemic review.

Study	Study design	Country	Patient number	Median age	Primary diagnosis	stage of GI–GVHD	Dose of vedolizumab (mg)	Use of vedolizumab
Chen et al., 2019a (19)	Cohort study	USA	3	58 yr (19–72)	MD (n=3; 14%); MP/MD (n=3; 14%); MD (n=2, 10%); AML (n=6; 29%); precursor lymphoid (n=3; 14%); others (n=2; 10%)	N/A (prophylactic use)	75	Prophylaxis
Chen et al., 2019b (19)	Cohort study	USA	21	22 yr (18–50)	AML (n=3; 100%)	N/A (prophylactic use)	300	Prophylaxis
Danylesko et al., 2018a (13)	RCT	Israel, Norway, Lithuania	13	55 yr (19–67)	AML (n=14; 48%); lymphoma (n=6; 21%); ALL (n=4; 14%); myelofibrosis (n=3; 10%); severe aplastic anemia (n=2; 7%)	stage 3 (n=3; 10%); stage 4 (n=26; 90%)	300	Second line treatment
Danylesko et al., 2018b (13)	RCT	Israel, Norway, Lithuania	16				300	Third line treatment
Mehta et al., 2020 (27)	Retrospective cohort	USA	9	44 yr (28–69)	MD (n=2; 22%); AML (n=4; 44%); ALL (n=1; 11%); others (n=2; 22%)	Stage 1 (n=1; 11%); stage 2 (n=2; 22%); stage 3 (n=3; 33%); stage 4 (n=3; 33%)	300	Second line treatment
Fløisand et al., 2016 (28)	Case series	N/A	6	48 yr (42–62)			300	First line and second line treatment
Fløisand et al., 2018 (12)	Retrospective review	Belgium, Norway, Sweden, and the USA	29	50 yr (16~69)	MP (n=1; 3%); MD/MP (n=2; 7%); MD (n=3; 10%); AML (n=8; 27%); precursor lymphoma (n=3; 10%); lymphoma (n=1; 3%); B–cell neoplasm (n=3; 10%); others (n=8; 27%)	Stage 1 (n=5; 17%); stage 2 (n=6; 21%); stage 3 (n=11; 38%); stage 4 (n=7; 24%)	300	Second line treatment
Jørgensen et al., 2021a (29)	RCT	Belgium, Norway, Sweden, and the USA	8	57 yr (34–74)	MD (n=2; 25%); AML (n=3; 38%); others (n=3; 38%)	Stage 1 (n=1; 13%); stage 2 (n=1; 13%); stage 3 (n=4; 50%); stage 4 (n=2; 25%)	300	Second line treatment
Jørgensen et al., 2021b (29)	RCT	Belgium, Norway, Sweden, and the USA	9		MD/MP (n=1; 11%); MD (n=2; 22%); AML (n=1; 11%); precursor lymphoma (n=1; 11%); others (n=3; 33%)	Stage 1 (n=3; 33%); stage 3 (n=4; 44%); stage 4 (n=2; 22%)	600	Second line treatment
Isshiki et al., 2022 (30)	Case series	Japan	3	2.55 yr (1.5~4.4)	CGD (n=1; 33%); NEMO deficiency (n=1; 33%); SCN (n=1; 33%)	Stage 2 (n=1; 33%); stage 3 (n=1; 33%); stage 4 (n=1; 33%)	177	Second line or third line treatment
Bukauskas et al., 2017 (18)	Case series	Lithuania	5	N/A	N/A	Stage 3 (n=5; 100%)	300	Third line treatment

AML, acute myeloid leukemia; MP, myeloproliferative syndrome; MD, myelodysplastic syndrome; CGD, chronic granulomatous disease; NEMO, NF-κB essential modulator; SCN, severe congenital neutropenia.

### Characteristics and quality assessment of the included studies

There was a total of 122 patients who developed GI-aGVHD following HSCT from the included eight included studies. Acute GVHD was diagnosed according to the Glucksberg–Seattle criteria (27) and clinically graded according to the modified Glucksberg criteria (27). The median age of patients across studies ranged from 2.55 to 59 years (**Table 1**).

Of seven studies that reported details on stages of GI-aGVHD in patients undergoing vedolizumab treatment for GVHD, a total of 47 patients (pooled rate = 47.95%) were of stage 4, 31 patients (pooled rate = 31.63%) were of stage 3, 10 patients (10.2%) were of stage 2, and 10 patients (10.2%) were of stage 1. In addition, patients in one study on vedolizumab for the prophylaxis of GI-aGVHD (19) did not have baseline GI-aGVHD. Accordingly, the seven (12, 13, 18, 28–31) studies on vedolizumab for the treatment of GI-aGVHD were included in the meta-analysis on the efficacy of vedolizumab, while the study (19) on prophylactic use of vedolizumab was not considered in the meta-analysis on the efficacy of vedolizumab.

The type of transplant was matched related donor (MSD) in 71 patients (pooled rate = 86.58%), mismatched related donor (MMRD) in eight adults (pooled rate = 9.75%), and haploidentical in three adults (pooled rate = 3.65%) across studies. Three studies (12, 18, 29) did not report details of the transplant type. Of three studies that reported details of conditioning regimens, 41 patients (pooled rate = 58.57%) used the non-myeloablative conditioning (NMA) regimen and 29 patients (pooled rate = 41.42%) used the myeloablative conditioning (MAC) regimen, while five studies (13, 18, 28, 29, 31) did not report details on the conditioning regimen (**Table 1**). Overall, three studies were assessed as good quality, and the rest were fair (**Table 2**).

**Table 2:** Quality assessment of the included studies.

Quality of included study			
Study	Quality of selection	Comparability	Outcomes of study participants
Chen et al., 2019 (19)	★★★	★★	★★★
Danylesko et al., 2018 (13)	★★★	★★	★★
Mehta et al., 2020 (27)	★★	★	★★★
Fløisand et al., 2016 (28)	★★	★	★★
Fløisand et al., 2018 (12)	★★	★	★★★
Jørgensen et al., 2021 (29)	★★★	★★	★★★
Isshiki et al., 2022 (30)	★★	★	★★
Bukauskas et al., 2017 (18)	★★	★	★★

We awarded stars (★) according to Newcastle-Ottawa Quality Assessment Form for Cohort Studies.

### Overall response to vedolizumab in patients with GI-aGVHD

The pooled overall response to vedolizumab indicated significant overall response rate (ORR) at 14 days (pooled ORR = 60.53%, pooled risk ratio, RR =14.14, 95% CI: 2.95–67.71), 28 days (pooled ORR = 50.00%, pooled RR = 7.36, 95% CI: 2.14–25.37), and 12 months (pooled ORR = 76.92%, pooled RR = 13.66, 95% CI: 3.50–53.35) with low heterogeneity across studies (Tau-square = 0.00; I-square = 0%) (**Figure 1**).

### Complete response to vedolizumab in patients with GI-aGVHD

**Figure 2:**
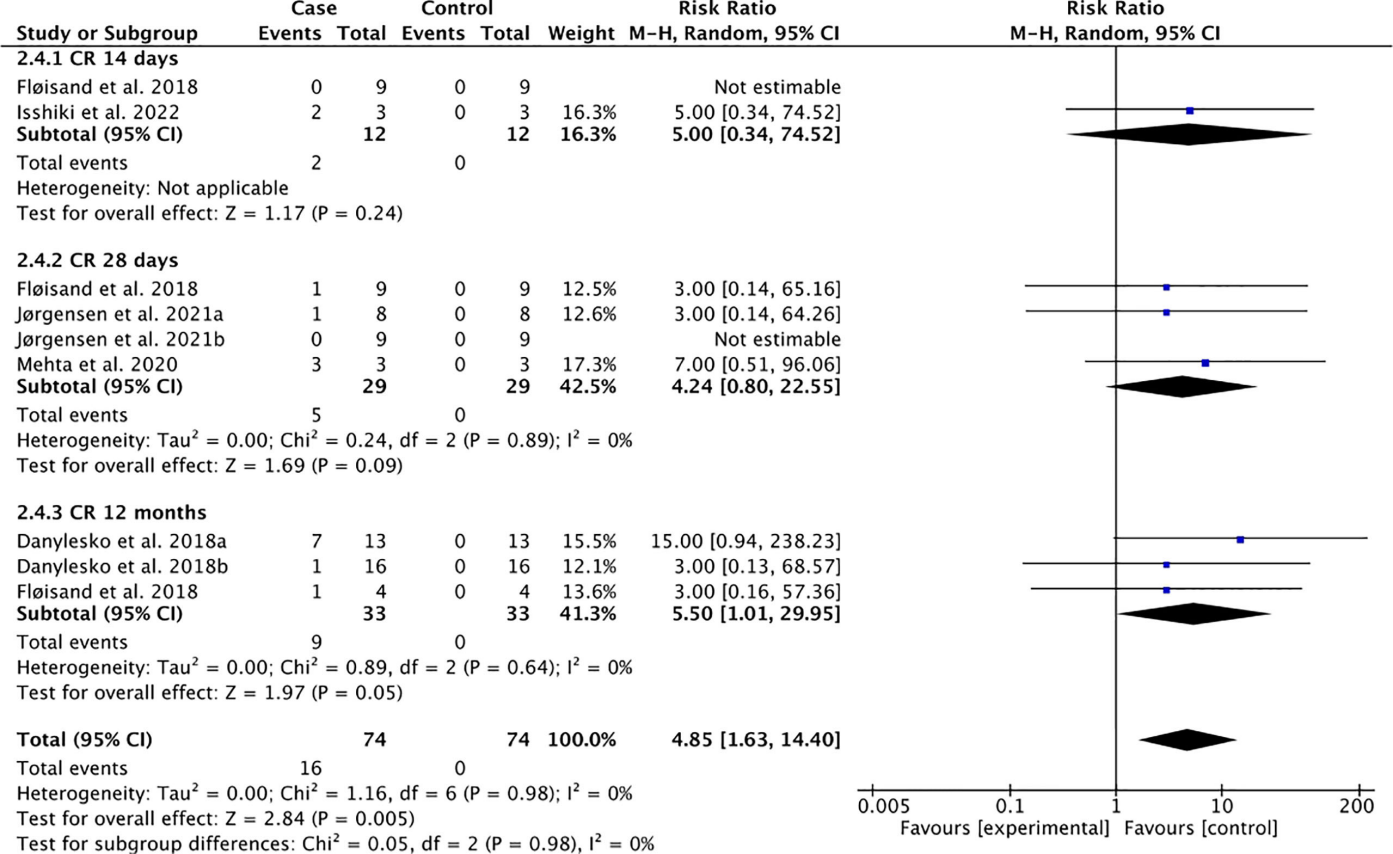
Complete response (CR) following the use of vedolizumab for GI-aGVHD.

Patients with GI-aGVHD showed a statistically significant complete response to vedolizumab at 12 months (pooled CRR = 27.27%, pooled RR = 5.50, 95% CI: 1.01–29.95) from baseline with low heterogeneity across studies (Tau-square = 0.00; I-square = 0%) (**Figure 2**). To the contrary, the pooled complete response at 14 days (pooled CRR = 16.67%, pooled RR = 5.00, 95% CI: 0.34–74.52) and at 28 days (pooled CRR = 17.24%, pooled RR = 4.24, 95% CI: 0.80–22.55) did not reach statistical significance (**Figure 2**). Collectively, these findings demonstrate possible long-term benefits of vedolizumab for patients with persistent GI-GVHD.

### Mortality following the use of vedolizumab in patients with GI-aGVHD

**Figure 3:**
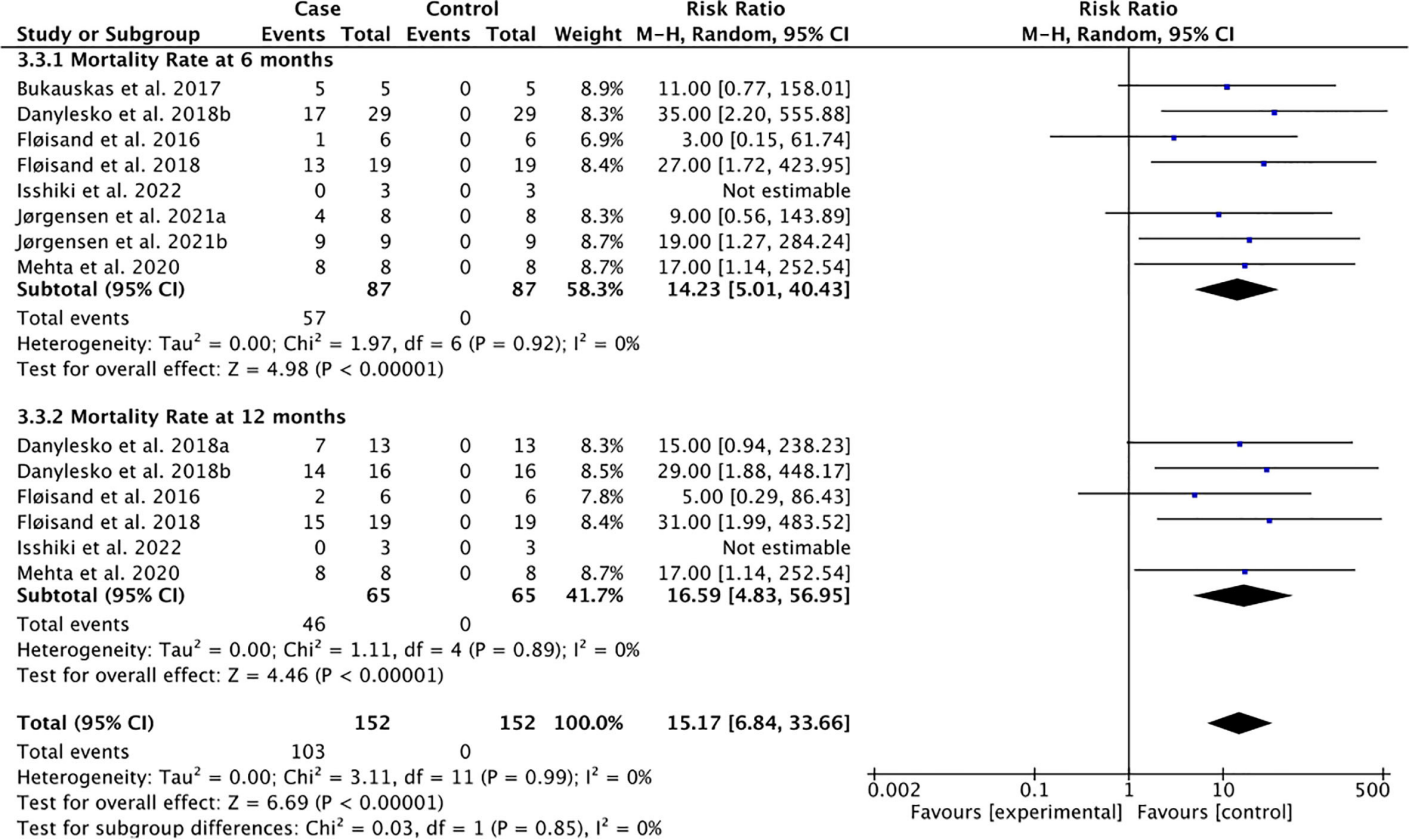
Mortality rate following the use of vedolizumab for GI-aGVHD.

The pooled mortality rate at 6 months (65.51%) and 12 months (70.76%) were derived from seven studies of 87 patients and six studies of 89 patients, respectively, with low heterogeneity (Tau-square = 0.00; I-square = 0%) (**Figure 3**).

### Causes of death following the use of vedolizumab in patients with GI-aGVHD

There were five studies (13, 19, 28–30) that recorded patients’ causes of death. The deaths were attributed to sepsis (n = 10), aGVHD (n = 7), the relapse of underlying diseases (n = 5), failure (n = 2), peritonitis (n = 1), thrombotic microangiopathy (n = 1), and mediastinitis (n = 1). 

### Treatment-related adverse events following the use of vedolizumab in patients with GI-aGVHD

**Figure 4:**
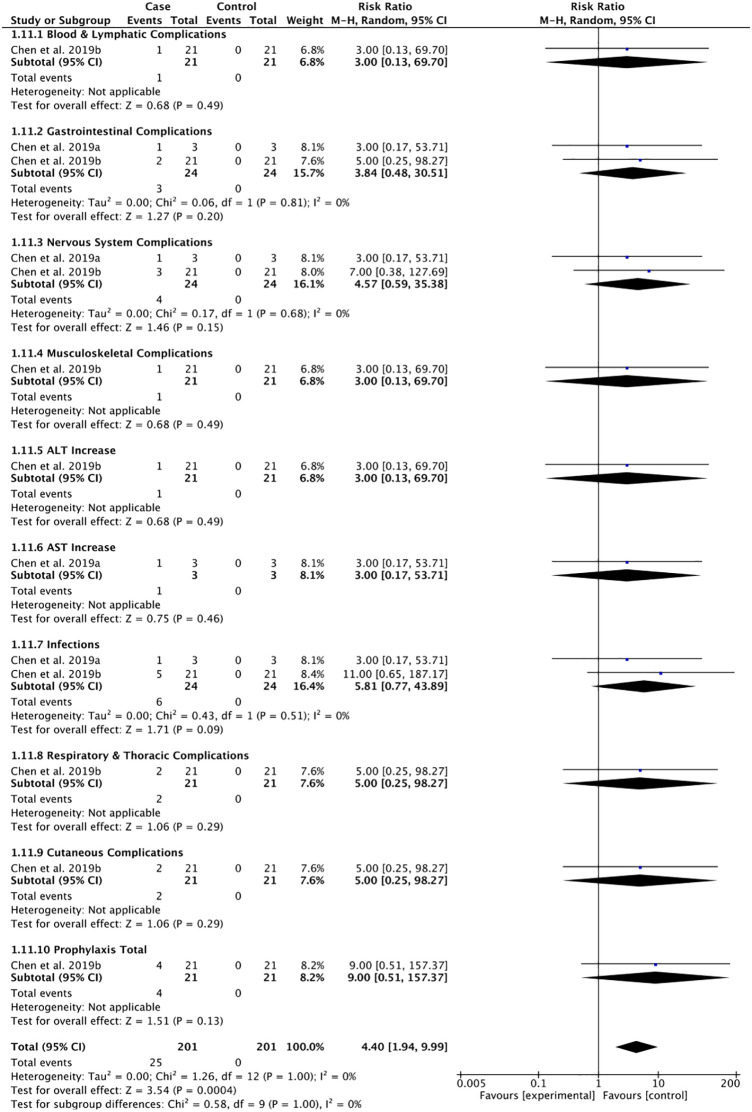
Adverse events following the use of vedolizumab to prevent GI-aGVHD.

**Figure 5:**
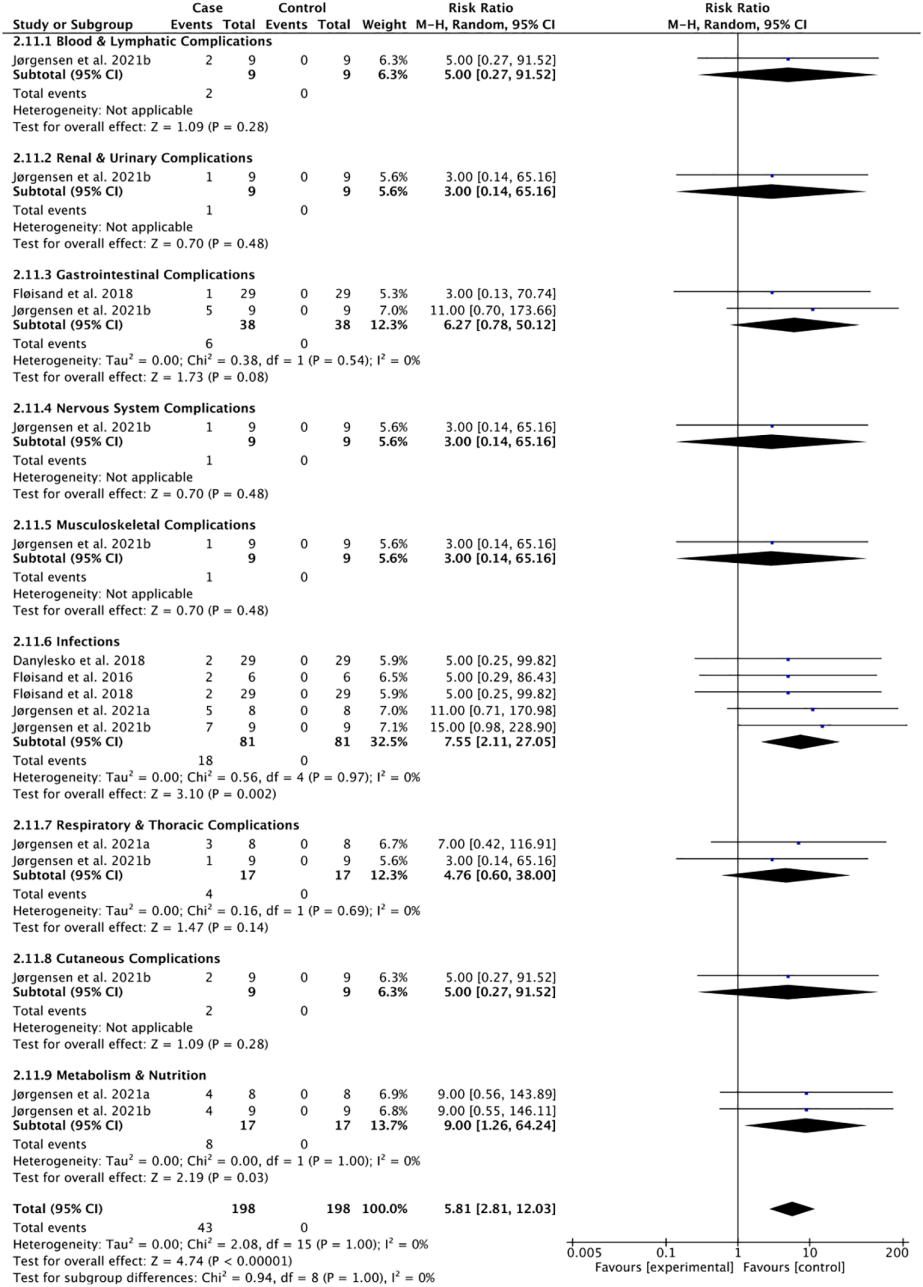
Adverse events following the use of vedolizumab to treat GI-aGVHD.

Pooled risks for adverse events including blood and lymphatic, renal and urinary, GI tract, nervous, musculoskeletal, hepatic, infectious, respiratory and thoracic, metabolic and nutritional, and cutaneous adverse events following prophylactic use of vedolizumab (**Figure 4**) and vedolizumab treatment for GI-aGVHD (**Figure 5**) were estimated in a total of 24 patients and 114 patients across studies, respectively. The risk of adverse events following prophylactic use of vedolizumab did not reach statistical significance (**Figure 4**), suggesting the safety of vedolizumab for the prevention of GI-aGVHD in patients undergoing HSCT. Despite the non-significant risk of most adverse events, the pooled risks for infections (pooled rate = 22.22% of 81 included participants, pooled RR = 7.55, 95% CI: 2.11–27.05) and metabolic and nutritional side effects (pooled rate = 47.06% of 17 included participants, pooled RR = 9.00, 95% CI: 1.26–64.24) were observed following the use of vedolizumab in patients with GI-aGVHD (**Figure 5**).

In reports of infections following the use of vedolizumab, one study (30) specified the numbers of each type of bacterial infections in patients with GI-aGVHD, with frequent pathogenic infections including *Enterococcus* (11.7%), *Escherichia* (11.1%), *Citrobacter* (11.11%), and cytomegalovirus (7.7%) infections. On the other hand, one study reported *Staphylococcus* infections in patients with GI-aGVHD (29). Stratifying the infected sites by organs (19, 30), it was pinpointed that the most common infections in patients with aGVHD were mediastinitis (11.1%), peritonitis (11.11%), pneumonia (6.8%), bronchitis (4.76%), cellulitis (4.76%), and mucosal infection (4.76%). Lastly, five out of 52 patients (9.6%) from three studies experienced sepsis (12, 29, 30). All in all, the risk of all treatment-related adverse events except infectious, metabolic, and nutritional complications following the use of vedolizumab in patients with GI-aGVHD did not reach statistical significance, providing the safety profile of vedolizumab for the treatment of GI-aGVHD.

## Discussion

The present study was the first systematic review and meta-analysis that assessed the safety and efficacy of vedolizumab for the prevention and the of GI-aGVHD. Results of clinical studies were comprehensively identified, evaluated, and summarized to determine the pooled efficacy of vedolizumab as an effective prophylaxis or therapeutic approach to GI-aGVHD. Despite experiencing adverse events including infections and metabolic or nutritional complications, patients on vedolizumab for the treatment of GI-aGVHD had significant overall response and complete response from baseline. While no significant adverse events were reported following prophylactic use of vedolizumab, more studies were warranted to reach a conclusive statement for its overall response rate and complete response rate and to address whether vedolizumab can be used for the standard prophylaxis of GI-aGVHD in patients undergoing allogenic HSCT.

As for the efficacy of vedolizumab for the treatment of GI-aGVHD, findings of the present meta-analysis demonstrated that the pooled overall response rate at 14 days, 28 days, and 12 months and pooled complete response at 12 months from baseline were evident, in which 70% of patients achieved an overall response on 12 months after first dose of vedolizumab. This finding was consistent with that reported by Coltoff et al. (28). In our findings, owing to the generally poor prognosis at baseline of GI-aGVHD, many patients on vedolizumab died in the first month of follow-up, while a remarkable clinical response was evident in patients who survived passing that time point. Furthermore, compared to the scenario in which vedolizumab was used as later-line treatments (13, 30), early administration of vedolizumab following steroid failure exerted a faster response and better overall response rate. Application of multiple immunosuppressive agents prior to the use of vedolizumab could result in microbiome imbalance, leading to unsatisfied prognosis following the use of later-line medications (32). At the same time, patients who used vedolizumab earlier were less likely to be immunosuppressed and develop subsequent infections (12, 13, 28). On the contrary, a few studies suggested poor overall outcomes following vedolizumab treatments in advanced aGVHD (13, 18, 30), which was explained by theories conjecturing that α4β7 integrins were no longer needed for the propagation of aGVHD after tissue injury and systemic injury occurred (13). In such theories, it was proposed that α4β7–MAdCAM-1 interactions were mainly involved in the early recruitment of T cells to the intestinal stem cell compartment (33). It warrants further studies to elucidate whether the expression of the α4β7 integrins can be a prognostic biomarker for GI-aGVHD and may be used to guide the use of vedolizumab in gastrointestinal inflammatory complications associated with GVHD. Another potential parameter for the early detection of GI-aGVHD is the calprotectin fecal level. Following the activation of leukocytes, calprotectin is released. Hence, the level of calprotectin is positively correlated to bowel inflammation. Moreover, it retains high stability and concentration under room temperature in feces, making it a reliable biomarker for GI inflammation detection aGVHD (34).

When it comes to whether vedolizumab can prevent GI-aGVHD in patients undergoing HSCT, there was one included clinical trial that addressed low risk of infection and no impairment of the graft-versus-tumor effect following the prophylactic use of vedolizumab (19). In particular, it was demonstrated that 300 mg intravenous vedolizumab in conjunction with tacrolimus and methotrexate (MTX) was well tolerated by adult patients who had undergone HSCT, among which only 12.5% of the participants receiving vedolizumab experienced lower-intestinal aGVHD and no dose-limiting toxicity was observed (19). Although there was limited evidence that supported vedolizumab as a standard prophylactic pharmacologic therapy for GVHD, the promising results of this study (19) enlightened the need for future studies that may ascertain the efficacy of prophylactic use of vedolizumab in patients receiving HSCT.

The present study provided the pooled safety profile of vedolizumab for the prophylaxis and treatment of GI-aGVHD, in which infections or metabolic and nutritional adverse events following vedolizumab as the second-line or above treatments were observed. This was in accordance with findings in a previous review that reported upper airway infections, nausea, and fatigue in over 2,000 patients on vedolizumab (35). Although it was suggested that theimmune suppressing effect of vedolizumab was local and was limited in the GI tract, which may not diminish immune responses to parenteral administered antigens (13, 36), still treatment-related infections were reported following the use of vedolizumab across studies. Nonetheless, the pooled rate of infections following vedolizumab treatment was lower than that following other systemic therapies. Compared with an infection rate of 59.09% following daclizumab and infliximab injection for GI-aGVHD (11), and an infection rate of 24% following the use of ruxolitinib for the treatment of GI-aGVHD (9), the present meta-analysis suggested a comparatively low pooled infection rate of 22% following vedolizumab treatment for GI-aGVHD. Furthermore, safety profiles in five out of eight included studies (12, 13, 19, 29, 30) specified the incidence of cytomegalovirus (CMV) infection following vedolizumab treatments. The mechanism of which may involve the preexisting inflammatory microenvironment in the bowel of patients with GI-aGVHD. To elaborate, CMV was dormant after its initial infection in most organs including the colon, while it can be reactivated upon inflammation or immunosuppression in the microenvironment (37, 38), for which the association between IBD and CMV infection had been suggested per extensively secreted proinflammatory cytokines in the bowel that caused the reactivation of CMV infection (39). Due to the shared immune-related pathogenesis between GI-aGVHD and IBD (14, 15, 40, 41), it was possible that CMV infections following vedolizumab treatments were attributed to the preexisting but not treatment-induced inflammatory microenvironment in GI-aGVHD.

Compared with a previous phase II trial which reported a 22±7% rate of developing aGVHD (grade II to IV) following the prophylactic use of CCR5 antagonists at 90 days after HSCT (42), the present meta-analysis showed a lower incidence of aGVHD (grade II to III) following the prophylactic use of vedolizumab (19.2%), with no participant experiencing grade IV aGVHD during the 6-month follow-up. Compared with the previously reported 12-month survival of 25% and 77% of overall response rate of 77% following pentostatin treatment in 12 patients with grade IV aGVHD (pooled rate = 60%) and eight patients with grade III aGVHD (pooled rate = 40%) (43), and another study demonstrating 12-month survival of 38% and overall response rate of 67% following the use of daclizumab and infliximab in 18 patients with grade IV aGVHD (pooled rate = 72%) and 7 patients with grade III aGVHD (pooled rate=28%) of grade III aGVHD (11), the present meta-analysis suggested a pooled 12-month overall survival of 29.23% and a pooled overall response rate of 76.92% following vedolizumab treatment in patients with stage 3 (31.63%) or stage 4 (47.95%) GI-aGVHD, for which the advantages of vedolizumab over pentostatin, daclizumab, and infliximab for advanced grades aGVHD were indicated. On the contrary, a retrospective cohort study (44) on the overall survival of 79 patients with SR-aGVHD using anti-thymocyte globulin (ATG) reported an estimated 6-month overall survival rate of 44%, while another phase III randomized clinical trial (45) comparing ATG with inolimomab found a 12-month overall survival rate of approximately 45% in all patients with aGVHD. The relatively higher mortality in the present meta-analysis than that in those studies (44, 45) was due mainly to the fact that most patients in the present study were of stage 3 or stage 4 GI-aGVHD at baseline, for which the more advanced grades with multiorgan involvement contributed to the worse prognosis (46–48). Overall, findings in the present study suggested that most deaths occurred in the first 6 months during the course of vedolizumab treatment. Juxtaposing the mortality profile and safety profile of vedolizumab for the prophylaxis and treatment of GI-aGVHD provided in the present study, it was suggested that patients should be closely monitored in the first 6 months after starting vedolizumab to prevent and to timely manage potential concomitant infections and metabolic or nutritional adverse events including decreased appetite, electrolyte imbalance, and hypoglycemia.

Limitations of the present meta-analysis included a lack of unified definition of SR-aGVHD across studies, which resulted in different criteria for the initiation of vedolizumab treatment. Second, due to the limited quantity of existing studies, not only clinical trials but also real-world studies and case series were included in the meta-analysis, which might compromise the external validity of our estimation on the effect of vedolizumab. Third, due to the limited sample size of each study, underlying conditions of aGVHD such as the primary disease, conditioning regimen, the status of human leukocyte antigen (HLA) matching, and comorbidities were not matched in the included studies, with which the lack of information might result in residual confounding bias. That said, since the heterogeneity across studies through all analyses in the present study was low, it was inferred that all abovementioned potential biases were optimally controlled.

In summary, the use of vedolizumab was safe and effective in patients with GI-aGVHD, especially when administered earlier in the disease course of GI-aGVHD. Further studies are warranted to elucidate its efficacy for the prophylaxis of GI-aGVHD in patients undergoing HSCT.

## Data availability statement

The raw data supporting the conclusions of this article will be made available by the authors, without undue reservation. 

## Author contributions

KSM and AA contribute to the conception and design of the study. ACWL, CD, and STT performed the literature search. ACWL and CD further completed the data screening and data extraction. ACWL analyzed the data and wrote the first draft of the manuscript. KSM created the tables and figures of this study. ACWL, CD, STT, AA, and KSM contributed to manuscript revision and read and approved the submitted version.

## Conflict of interest

The authors declare that the research was conducted in the absence of any commercial or financial relationships that could be construed as a potential conflict of interest.

## Publisher’s note

All claims expressed in this article are solely those of the authors and do not necessarily represent those of their affiliated organizations, or those of the publisher, the editors and the reviewers. Any product that may be evaluated in this article, or claim that may be made by its manufacturer, is not guaranteed or endorsed by the publisher.
