# Supplementary figures and images for: Vedolizumab for acute gastrointestinal graft-versus-host disease: A systematic review and meta-analysis
Source: Front Immunol. 2022 Nov 11;13:1025350. doi: 10.3389/fimmu.2022.1025350 (PMC9692080; doi:10.3389/fimmu.2022.1025350)

## PRISMA flow diagram for inclusions of studies

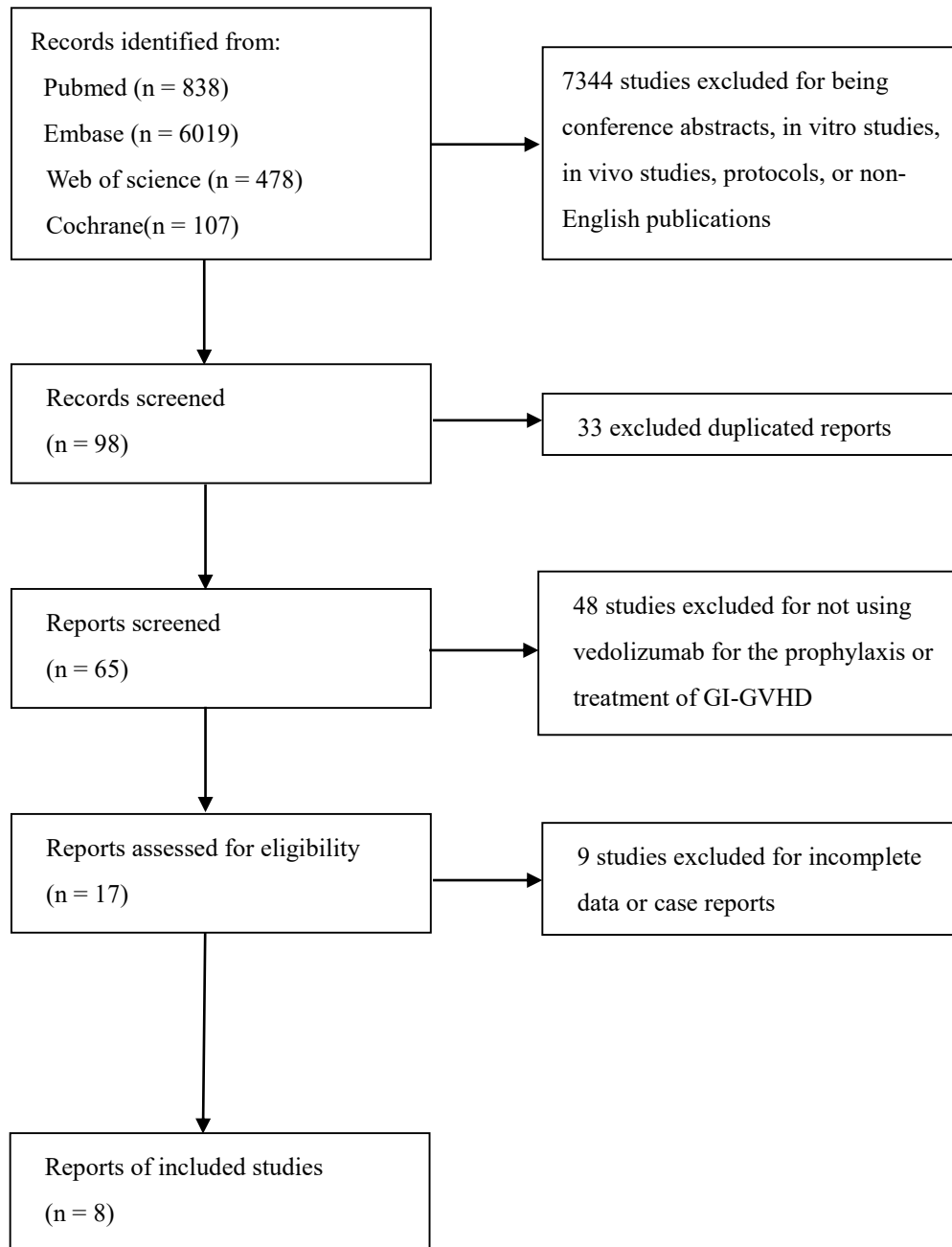

Supplement: Supplementary file 1 [file Image_1.pdf]
